# The impact of short video addiction on the creativity of college students: the mediating role of intrinsic motivation and innovative self-efficacy

**DOI:** 10.3389/fpsyg.2026.1835206

**Published:** 2026-05-21

**Authors:** Pengfei Sha, Yongzhen Gu

**Affiliations:** 1 School of Materials Science and Engineering, Changzhou University, Changzhou, China; 2 School of Materials Science and Engineering, Changzhou Vocational Institute of Industry Technology, Changzhou, China

**Keywords:** chain mediation, creativity, innovative self-efficacy, intrinsic motivation, short video addiction

## Abstract

**Objective:**

This study aimed to investigate the relationship between short video addiction and creativity among college students, and to examine the mediating roles of intrinsic motivation and innovative self-efficacy in this relationship.

**Methods:**

A questionnaire survey was conducted from October to November 2025 among 555 college students recruited from multiple universities in Jiangsu Province. The Short Video Addiction Scale, Intrinsic Motivation Scale, Innovative Self-Efficacy Scale, and College Student Creativity Scale were administered. Data were analyzed using SPSS 26.0 and AMOS 28.0, involving descriptive statistics, correlation analysis, and structural equation modeling. The chain mediation effect was tested using the PROCESS macro.

**Results:**

(1) Short video addiction was significantly negatively correlated with college students’ creativity (*β* = −0.210, *p* < 0.001). (2) Short video addiction significantly and negatively predicted intrinsic motivation (*β* = −0.285, *p* < 0.001) and innovative self-efficacy (*β* = −0.187, *p* < 0.001), whereas intrinsic motivation and innovative self-efficacy each significantly and positively predicted creativity (*β* = 0.504, *p* < 0.001; *β* = 0.353, *p* < 0.001). (3) The mediating effect of intrinsic motivation on the relationship between short video addiction and creativity was significant, with an effect value of −0.113, accounting for 26.59% of the total effect. (4) The mediating effect of innovative self-efficacy was also significant, with an effect value of −0.064, accounting for 15.06% of the total effect. (5) Intrinsic motivation and innovative self-efficacy played a chain mediating role between short video addiction and creativity, with an effect value of −0.035, accounting for 8.24% of the total effect.

**Conclusion:**

Short video addiction is negatively associated with college students’ creativity, and this association may operate both directly and indirectly through diminished intrinsic motivation and innovative self-efficacy. These two factors constitute a “motivation → belief” chain mediating pathway. This study identifies potential psychological mechanisms linking short video addiction to college students’ creativity, offering a theoretical basis and practical implications for mental health education and the cultivation of innovative talents in higher education institutions.

## 1 Introduction

In the contemporary context where digital technologies are deeply integrated into daily life, emerging media platforms, represented by short videos, have rapidly captured the attention of college students through fragmented content, diverse formats, and algorithm-driven precise distribution mechanisms, becoming an important vehicle for information acquisition, entertainment, and social interaction (Wang and Yao, 2024). However, while satisfying users’ immediate psychological needs, this highly convenient and interactive media form also harbors the potential risk of inducing excessive use and even behavioral addiction. Short video addiction, defined as a phenomenon where individuals, unable to effectively control their viewing behavior, become immersed in short video platforms for extended periods, leading to time mismanagement, increased psychological dependence, and impaired social functioning, has become a psychological and behavioral issue of significant concern in the digital age (Li T. et al., 2025). Particularly for college students, who are in a critical period of rapid physical and mental development, personality shaping, and value formation, the potential harm of short video addiction should not be underestimated. Concurrently, creativity, as a core component of the essential competencies for talents in the 21st century, refers to an individual’s ability to generate ideas, products, or solutions that are both novel and useful. It holds significant strategic importance for the enhancement of college students’ academic abilities, their career development planning, and even the cultivation of national innovative capacity (Khalil et al., 2023). The university stage is generally regarded as the peak period for the development of individuals’ cognitive abilities and innovative potential. Therefore, exploring the facilitating factors and inhibiting mechanisms that influence the development of college students’ creativity has consistently remained an important topic in psychology, pedagogy, and even sociological research.

While existing research has begun to focus on the impact of digital media use on individual cognitive functions, most discussions remain at the level of general screen time or social media usage. Few studies have systematically investigated the intrinsic logical connection between this specific behavioral pattern of short video addiction and higher-order cognitive abilities—particularly creativity—and the underlying mechanisms of its effect remain largely unexplored. The “short, fast, and immediate” information presentation mode and instant feedback mechanisms of short video platforms may subtly influence individuals’ attentional stability, deep processing capacity, and intrinsic motivation, thereby affecting the development of creative thinking (Chen et al., 2022). This potential influence pathway urgently requires theoretical construction and empirical testing. Grounded in Self-Determination Theory and Social Cognitive Theory, this study aims to explore the mechanism by which short video addiction affects college students’ creativity, introducing intrinsic motivation and innovative self-efficacy as key mediating variables to construct a hierarchical chain mediation model. Intrinsic motivation emphasizes the drive for individuals to engage in creative activities due to interest, enjoyment, or inherent satisfaction, while innovative self-efficacy refers to an individual’s belief in their ability to produce innovative outcomes (Gu et al., 2015). These two variables, operating at the levels of motivational source and belief support respectively, may play a mediating role in the relationship between short video addiction and creativity. By systematically investigating “how” and “why” short video addiction affects creativity, this study seeks to provide theoretical support for understanding the dual effects of the digital media environment on college students’ cognitive development, and to offer evidence-based foundations for mental health education, the cultivation of innovative talents, and digital literacy interventions in higher education institutions.

### 1.1 The relationship between short video addiction and creativity

In the contemporary era of digital proliferation, short video platforms are profoundly altering the information reception patterns and cognitive habits of the youth demographic (Xu et al., 2023). For college students, who are in a critical period of cognitive development and personality formation, short video addiction has emerged as a potential risk factor influencing their higher-order thinking abilities, particularly the development of creativity. From a cognitive psychology perspective, the generation of creativity relies on deep information processing and cognitive flexibility, both of which are supported by sufficient cognitive resources (Otto, 2025). According to the theory of limited cognitive resources, an individual’s cognitive processing capacity possesses clear limitations. Through high-intensity sensory stimulation, short video addiction continuously occupies the attention of college students. Consequently, the cognitive resources intended for deep learning and reflection are substantially depleted, hindering their ability to enter the focused state necessary for innovative thinking and thereby inhibiting the generation and expression of creative ideas (Xie et al., 2023). Of further concern is that short video addiction can also alter an individual’s cognitive processing style: the “fragmented” content presentation and “instant gratification” feedback mechanisms of short videos gradually cultivate a jumping and superficial cognitive style. This weakens individuals’ sustained attention and deep analytical abilities, which fundamentally conflicts with the coherence, expansiveness, and depth of reflection required for creativity (Ye et al., 2025). Neuroscientific research further indicates that the high-frequency stimulation from short videos can over-activate the brain’s reward system, fostering a dependence on behaviors with low cognitive load and leading individuals to actively avoid creative tasks that necessitate deep thought (Hong et al., 2026). It is evident that short video addiction not only occupies students’ time but also subtly reshapes their value orientations and willingness to exert effort. Existing empirical research has explicitly revealed a negative association between problematic mobile phone use and creativity, indirectly suggesting that short video addiction may significantly inhibit the innovative potential of college students. Based on the aforementioned theoretical analysis and empirical research foundation, the following hypothesis is proposed: Hypothesis 1: Short video addiction exerts a significant negative predictive effect on the creativity of college students.

### 1.2 The mediating role of intrinsic motivation

Intrinsic motivation is a crucial psychological force that drives individuals to engage in creative activities, fundamentally characterized by active involvement due to interest and enjoyment in the activity itself, rather than reliance on external rewards or pressure (Zhang et al., 2020). According to Self-Determination Theory, the generation and maintenance of intrinsic motivation depend on the satisfaction of three basic psychological needs: autonomy, competence, and relatedness. When individuals perceive freedom of choice in an activity, experience a sense of capability in meeting challenges, and feel genuine connections with others, intrinsic motivation is activated and strengthened (Karimi and Sotoodeh, 2019). However, short video addiction may erode the pathways through which these three basic psychological needs are satisfied, thereby diminishing the level of intrinsic motivation in college students. This is manifested in three specific aspects: First, it deprives the need for autonomy. Addicted individuals fall into a state of “passive scrolling,” where viewing behavior is manipulated by algorithms, making it difficult to autonomously control viewing duration and content type, thus damaging the need for autonomy (Zhang et al., 2024). Second, it weakens the need for competence. Watching short videos is a unidirectional, passive process of information reception. Activities with low cognitive load rarely provide experiences of achievement and cannot offer the challenge and sense of mastery required for feelings of competence (Kuykendall et al., 2020). Third, it superficializes the need for relatedness. Social interactions on short video platforms tend to be shallow and cannot replace the emotional resonance and deep support found in real-life interpersonal relationships, leaving students’ need for relatedness largely unfulfilled (Zhu et al., 2024). When these three basic psychological needs are deficient, an individual’s intrinsic interest in academic exploration and creative activities subsequently declines. This decline in intrinsic motivation can strip individuals of their ability to derive pleasure from the creative process, which is precisely the internal drive that creativity relies upon. Individuals with high intrinsic motivation can experience enjoyment and a sense of value in creative activities, proactively investing more cognitive resources; conversely, college students with insufficient intrinsic motivation tend to prefer effortless entertainment for instant gratification rather than proactively engaging in creative endeavors (Urban et al., 2025). Therefore, short video addiction may undermine intrinsic motivation, thereby indirectly inhibiting the creative performance of college students. Based on the above analysis, the following hypothesis is proposed: Hypothesis 2: Intrinsic motivation mediates the relationship between short video addiction and college students’ creativity.

### 1.3 The mediating role of innovative self-efficacy

Innovative self-efficacy, defined as an individual’s judgment of their capability to accomplish specific creative tasks, constitutes a crucial cognitive mechanism linking media usage behavior to creative performance. Self-efficacy reflects an individual’s confidence in their own abilities and influences their behavioral choices when facing challenges (Choi et al., 2021). Individuals with high innovative self-efficacy demonstrate greater resilience when confronted with complex tasks, whereas those with low innovative self-efficacy tend to experience self-doubt and avoid problems (Ren and Shen, 2024). Short video addiction may undermine the innovative self-efficacy of college students through several pathways. Firstly, short video addiction diminishes a core source of self-efficacy: enactive mastery experiences. When college students spend excessive time watching short videos, opportunities for active exploration and creative output are reduced. This not only hinders the accumulation of successful innovation experiences but also reinforces negative evaluations of their own capabilities (Lin et al., 2023). Secondly, vicarious experiences and verbal persuasion are adversely affected. The entertaining and fragmented nature of content on short video platforms makes it difficult to provide effective innovative role models. Furthermore, addiction to short videos reduces students’ positive communication with teachers and peers, thereby weakening the external support essential for enhancing self-efficacy (Xu et al., 2026). Thirdly, the negative emotional states resulting from excessive short video use can impact self-efficacy. Such a negative psychological background makes individuals more prone to self-doubt when facing creative tasks. Negative emotions and low efficacy beliefs mutually reinforce each other, potentially leading to a stable cognition of “I am not good at innovation” (Li S. et al., 2025). When innovative self-efficacy is persistently low, individuals tend to avoid challenges in creative situations, give up prematurely, and rely heavily on external guidance. The belief that “I am not capable of innovation” can ultimately constrain their actual creative performance (Thundiyil et al., 2016). Based on the preceding analysis, the following hypothesis is proposed: Hypothesis 3: Innovative self-efficacy mediates the relationship between short video addiction and college students’ creativity.

### 1.4 The chain mediating role of intrinsic motivation and innovative self-efficacy

Within the mechanism through which short video addiction affects college students’ creativity, intrinsic motivation and innovative self-efficacy do not function merely as parallel pathways; a progressive relationship may exist between them. From the perspective of the psychological process underlying creative behavior, intrinsic motivation provides the initial driving force, determining “willingness to engage,” whereas innovative self-efficacy influences the sustainability and resilience of action, determining “ability to persist.” This “motivation → belief” sequence constitutes a deep psychological pathway through which short video addiction inhibits creativity (Chen et al., 2024). The mechanism by which short video addiction erodes this pathway is twofold. First, it directly diminishes the level of intrinsic motivation in college students. When substantial time is devoted to consuming short videos, individuals’ intrinsic interest in learning and creative activities declines, leading to a loss of the psychological drive to participate in innovative endeavors, with “unwillingness to do” becoming the primary obstacle (Zhao and Kou, 2024). Second, it obstructs the formation pathway of innovative self-efficacy. According to Social Cognitive Theory, successful experiences require active engagement and practical exploration. By reducing intrinsic motivation, short video addiction leads college students to decrease their creative attempts, thereby losing the empirical foundation necessary for acquiring the belief “I am capable of innovation.” The decline in intrinsic motivation results in fewer practice opportunities, depriving innovative self-efficacy of its essential support (Ye et al., 2024). Furthermore, the sense of immersion and pleasure derived from intrinsic motivation can reinforce self-efficacy beliefs; failure to obtain such intrinsic satisfaction inhibits positive evaluations of one’s own abilities. Therefore, short video addiction erodes intrinsic motivation, leading to a lack of drive; this lack of drive results in a scarcity of innovative practice; this scarcity prevents individuals from validating their abilities, ultimately leading to low innovative self-efficacy. The rupture of this “motivation → belief” chain elucidates the dual psychological mechanism through which short video addiction inhibits college students’ creativity (Yan, 2022). Although existing research has explored the roles of intrinsic motivation and innovative self-efficacy in creativity formation, systematic empirical testing integrating both as a chain mediation model to reveal the complete psychological pathway through which digital media addiction affects creativity remains lacking. Based on the aforementioned analysis, the following core hypothesis is proposed: Hypothesis 4: Intrinsic motivation and innovative self-efficacy play a chain mediating role in the relationship between short video addiction and college students’ creativity.

**Figure 1:**
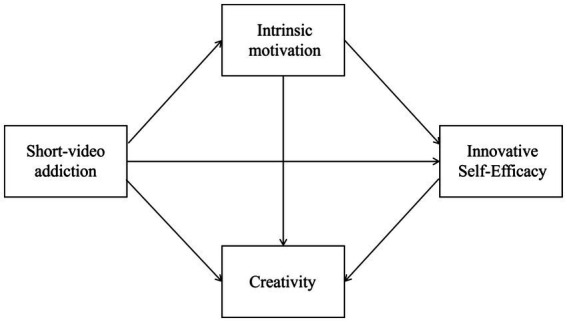
Proposed chain mediation model.

In summary, grounded in Self-Determination Theory and Social Cognitive Theory, the present study aims to investigate the relationship between short video addiction and creativity among college students, as well as the mediating roles played by intrinsic motivation and innovative self-efficacy in this relationship. To this end, a conceptual framework was constructed, as illustrated in Figure 1.

## 2 Methods

### 2.1 Participants

College students enrolled in several higher education institutions in Jiangsu Province were recruited as participants for this study. An electronic questionnaire was generated using the Questionnaire Star platform and was distributed online via links or Quick Response (QR) codes on the WeChat platform for data collection. Prior to questionnaire administration, all 555 college students who participated in the survey read and provided informed consent on the first page of the questionnaire, which covered the research purpose, data usage, anonymity assurance, and the right to voluntarily withdraw. A total of 612 questionnaires were collected. During the data cleaning phase, questionnaires were excluded based on the following criteria: (1) patterned responses, such as consistently selecting the same option (e.g., all answers “3”), zigzag patterns, or periodic repetitions; (2) excessively short completion times (less than 120 s) or excessively long completion times (more than 900 s). Following a meticulous case-by-case review based on these standards, 57 invalid questionnaires were removed, resulting in a final sample of 555 valid responses. The effective response rate was 90.69%. Within the valid sample, 379 participants (68.3%) were male and 176 (31.7%) were female. Regarding grade level, 158 students (28.5%) were freshmen, 142 (25.6%) were sophomores, 136 (24.5%) were juniors, and 119 (21.4%) were seniors.

### 2.2 Measures

#### 2.2.1 Short video addiction

Short video addiction was measured using the Short Video Addiction Scale developed by Qin et al. (2019). This scale consists of 14 items and encompasses four dimensions: loss of control, withdrawal symptoms, escapism, and inefficiency. A sample item from the scale is: “I find myself spending more time watching short videos than I had intended.” All items were rated on a 5-point Likert scale, with responses ranging from 1 (strongly disagree) to 5 (strongly agree). The total score was calculated by summing the scores of all items, with higher scores indicating a more pronounced tendency toward short video addiction. In the present study, the Cronbach’s *α* coefficient for the total scale was 0.863.

#### 2.2.2 Intrinsic motivation

Intrinsic motivation was assessed using a scale developed based on the theoretical framework proposed by Amabile (1985) and related preliminary studies. This scale comprises six items designed to measure students’ level of intrinsic motivation in academic activities. Sample items include: “I enjoy generating innovative ideas for course projects or academic research” and “I am passionate about improving my learning techniques and research methods, striving for continuous improvement.” All items were rated on a 7-point Likert scale, with responses ranging from 1 (strongly disagree) to 7 (strongly agree). In the present study, the Cronbach’s *α* coefficient for this scale was 0.859, indicating good internal consistency reliability.

#### 2.2.3 Innovative self-efficacy

Innovative self-efficacy was measured using the Innovative Self-Efficacy Scale developed by Tierney and Farmer (2002). This scale is designed to assess an individual’s confidence in their ability to successfully engage in innovative activities. The scale consists of four items, with sample items including: “I feel that I am good at generating novel ideas” and “I have a knack for further developing and refining the ideas of others.” These items effectively capture individuals’ self-efficacy beliefs regarding innovation and creative tasks. All items were rated on a 7-point Likert scale, with responses ranging from 1 (strongly disagree) to 7 (strongly agree). In the present study, the Cronbach’s *α* coefficient for this scale was 0.869.

#### 2.2.4 Creativity

Creativity was measured using the College Student Creativity Scale developed by He et al. (2015). This scale consists of 16 items and encompasses three dimensions: divergent thinking, application of intelligence, and personality characteristics. All items were rated on a 5-point Likert scale, with responses ranging from 1 (strongly disagree) to 5 (strongly agree). The total score was calculated by summing the scores of all items, with higher scores indicating a higher level of creativity. In the present study, the Cronbach’s α coefficient for this scale was 0.853.

### 2.3 Statistical analysis

Data analysis was conducted using IBM SPSS 26.0 for descriptive statistics, reliability analysis, and correlation analysis. Structural equation modeling was performed using Amos 28.0 to construct the measurement and structural models and to test the model fit. For the mediation analysis, Model 6 of the PROCESS macro was utilized. Statistical significance testing and path coefficient estimation were conducted using the bootstrap method with 5,000 resamples. A mediation effect was considered statistically significant if its 95% confidence interval did not include zero. Additionally, *p*-values less than 0.05 were considered indicative of statistical significance.

## 3 Results

### 3.1 Common method bias test

To examine the potential presence of common method bias, Harman’s single-factor test was conducted. The results revealed that three factors with eigenvalues greater than 1 were extracted. The first factor accounted for 38.52% of the total variance, which is below the recommended threshold of 40% (Podsakoff et al., 2003). These findings suggest that common method bias was not a serious concern in this study.

### 3.2 Descriptive statistics and correlation analysis

Correlation analysis revealed that short video addiction was significantly negatively correlated with intrinsic motivation, innovative self-efficacy, and creativity (*p* < 0.01). Conversely, intrinsic motivation was significantly positively correlated with both innovative self-efficacy and creativity (*p* < 0.01). Furthermore, a significant positive correlation was observed between innovative self-efficacy and creativity (*p* < 0.01). The means, standard deviations, and correlation coefficients for all study variables are presented in Table 1.

**Table 1:** Mean, standard deviation, and correlation analysis.

Variable	*M*	SD	1	2	3	4
1. Short video addiction	2.452	0.778	–			
2. Intrinsic motivation	2.426	0.836	−0.245**	–		
3. Innovative Self-Efficacy	2.435	0.792	−0.276**	0.439**	–	
4. Creativity	2.443	0.825	−0.400**	0.632**	0.582**	–

M, mean; SD, standard deviation. *Correlation is significant at the 0.05 level; **Correlation is significant at the 0.01 level.

### 3.3 Structural equation modeling and model fit

In this study, the latent variables examined included short video addiction, intrinsic motivation, innovative self-efficacy, and creativity. Short video addiction was specified as the independent variable, creativity as the dependent variable, and intrinsic motivation and innovative self-efficacy as mediating variables. A structural equation model was constructed using Amos 28.0 to test the hypothesized relationships, and the model fit was evaluated. The fit indices are presented in Table 2. According to the criteria recommended by Hu and Bentler (1998) and McDonald and Ho (2002), a model is considered to have a good fit when the Comparative Fit Index (CFI) and Tucker–Lewis Index (TLI) are greater than 0.90, and the Standardized Root Mean Square Residual (SRMR) and Root Mean Square Error of Approximation (RMSEA) are less than 0.08. The results of the present study indicated a good model fit: *χ*^2^ = 238.92, df = 123, RMSEA = 0.052, SRMR = 0.038, TLI = 0.976, CFI = 0.981. These indices suggest that the hypothesized model fits the data well.

**Table 2:** Model fit indices.

Indices	*X* ^2^	df	RMSEA	SRMR	TLI	CFI
Index value	238.92	123	0.052	0.038	0.976	0.981

### 3.4 Mediation effect test

**Figure 2:**
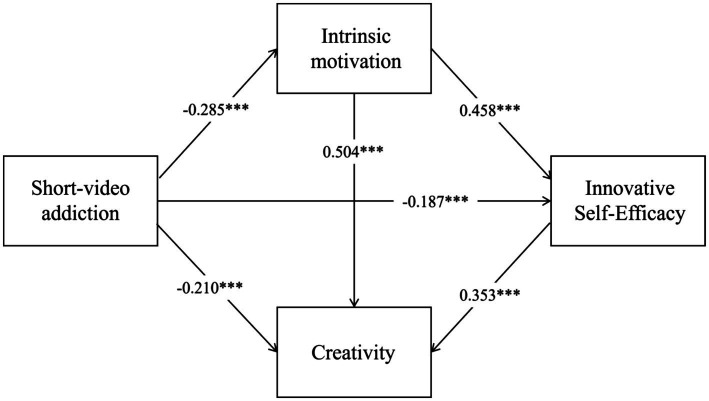
Standardized path coefficients of the chain mediation model. ***p < 0.001.

The path effects of short video addiction, intrinsic motivation, and innovative self-efficacy on creativity are illustrated in Figure 2. Short video addiction exerted a significant negative predictive effect on creativity (*β* = −0.210, *p* < 0.001), thereby supporting Hypothesis 1. A significant negative correlation was found between short video addiction and intrinsic motivation (*β* = −0.285, *p* < 0.001), while intrinsic motivation was significantly positively correlated with creativity (*β* = 0.504, *p* < 0.001). These findings not only support Hypothesis 2 but also suggest that short video addiction can indirectly influence creativity through the mediating variable of intrinsic motivation. Furthermore, short video addiction was significantly negatively correlated with innovative self-efficacy (*β* = −0.187, *p* < 0.001), and innovative self-efficacy was significantly positively correlated with creativity (*β* = 0.353, *p* < 0.001), indicating that short video addiction can indirectly affect creativity through innovative self-efficacy, thus supporting Hypothesis 3. Additionally, a significant positive correlation was observed between intrinsic motivation and innovative self-efficacy (*β* = 0.458, *p* < 0.001). This suggests that short video addiction can indirectly influence creativity through the chain mediating effect of intrinsic motivation and innovative self-efficacy, thereby confirming Hypothesis 4.

The results of the correlation and path analyses indicated significant relationships among the variables, suggesting that short video addiction exerts both direct and indirect effects on college students’ creativity. This pattern of relationships was further examined using the bootstrap method. Mediation analysis was conducted using Model 6 of the PROCESS macro (Hayes, 2017), employing a non-parametric percentile bootstrap method with 5,000 resamples and 95% confidence intervals (CIs). The results are presented in Table 3. For the first indirect path (short video addiction → intrinsic motivation → creativity), the 95% confidence interval was [−0.157, −0.074]. As this interval did not contain zero, the mediating effect was considered statistically significant. This indirect effect accounted for 26.59% of the total effect, indicating that more than one-quarter (26.59%) of the negative impact of short video addiction on college students’ creativity was mediated through the diminishment of intrinsic motivation. For the second indirect path (short video addiction → innovative self-efficacy → creativity), the 95% confidence interval was [−0.094, −0.037]. This interval also excluded zero, confirming a significant mediating effect, which accounted for 15.06% of the total effect. For the chain mediating path (short video addiction → intrinsic motivation → innovative self-efficacy → creativity), the 95% confidence interval was [−0.049, −0.022]. Given that zero was not included in this interval, the chain mediating effect was deemed significant. This indirect effect accounted for approximately 8.24% of the total effect.

**Table 3:** Mediating effect analysis.

Content	Path relationship	Effect size	Boot LL CI	Boot UL CI
Indirect path 1	Short video addiction → Intrinsic motivation → Creativity	−0.113	−0.157	−0.074
Indirect path 2	Short video addiction → Innovative Self-Efficacy → Creativity	−0.064	−0.094	−0.037
Chain mediation	Short video addiction → Intrinsic motivation → Innovative Self-Efficacy → Creativity	−0.035	−0.049	−0.022
Direct effect	Short video addiction → Creativity	−0.213	−0.275	−0.150
Total effect		−0.425	−0.506	−0.343

## 4 Discussion

Grounded in Self-Determination Theory and Social Cognitive Theory, a chain mediation model was constructed in this study to investigate the mechanism by which short video addiction affects creativity among college students, with a particular focus on the mediating roles of intrinsic motivation and innovative self-efficacy. All proposed hypotheses were supported by the findings. The results revealed that short video addiction not only directly and negatively predicted college students’ creativity but also indirectly inhibited their creative performance by diminishing intrinsic motivation and innovative self-efficacy. Furthermore, these two mediators constituted a “motivation → belief” chain mediating pathway within the model. These findings extend the application of relevant theories to the understanding of the relationship between digital media use and creativity. A novel explanatory framework is provided for comprehending how short video addiction influences creativity through motivational and belief-based pathways. Empirical support is also offered for the hierarchical relationship between motivational and cognitive factors in the development of creativity. The results offer practical implications for mental health education and creativity cultivation in higher education institutions. It is suggested that attention should be paid to students’ short video usage behaviors, and guidance should be provided to help them develop healthy digital media habits. Simultaneously, emphasis should be placed on stimulating intrinsic motivation and enhancing innovative self-efficacy to promote the healthy development of their creativity. Furthermore, these findings provide valuable insights for family education and societal media literacy education.

### 4.1 Short video addiction and creativity

A significant negative predictive effect of short video addiction on college students’ creativity was found in this study, a result that is highly consistent with existing research. With the rapid development of short video platforms, college students have become one of the most frequent user groups of short videos, and the issue of addiction among this population has become increasingly prominent. Short video addiction is primarily characterized by a strong dependence on short video content, an inability to control usage time, and even disruption of normal academic and daily routines (Liao, 2024). From the perspective of cognitive resource theory, creativity, as a higher-order cognitive ability, relies on individuals’ capacity for deep information processing, associative integration, and reflective thinking, all of which require sufficient attention and time (Reche and Perfectti, 2020). Short video addiction occupies a substantial portion of individuals’ cognitive resources, hindering their ability to maintain the focused state necessary for deep thought, thereby inhibiting divergent thinking and creative expression (Lu et al., 2022). Furthermore, the fragmented nature of short video content habituates individuals to rapidly shifting attention, making it difficult to form coherent chains of thought. Once this cognitive habit becomes entrenched, it may exert a long-term negative impact on the development of creative thinking (Walla and Zheng, 2024). From a neuropsychological perspective, the immediate feedback mechanism of short videos activates the brain’s reward system, prompting individuals to pursue instant gratification. Creativity, however, often requires delayed gratification and sustained effort, representing a fundamental conflict between the two (Li Y. et al., 2025). Therefore, short video addiction is not merely a behavioral dyscontrol; it may erode the foundation of college students’ creativity at multiple levels, including cognitive structure, motivational orientation, and neural adaptation.

### 4.2 The mediating role of intrinsic motivation

The mediating role of intrinsic motivation in the relationship between short video addiction and creativity also warrants attention. The findings of this study indicate that short video addiction exerts a significant negative predictive effect on intrinsic motivation, while intrinsic motivation, in turn, has a significant positive predictive effect on creativity. This suggests that short video addiction can indirectly inhibit college students’ creativity by diminishing their intrinsic motivation. These results provide new empirical support for Self-Determination Theory. According to Self-Determination Theory, the generation of intrinsic motivation depends on the satisfaction of three fundamental psychological needs: the need for autonomy, the need for competence, and the need for relatedness. When college students engage in excessive short video use, their time management often becomes dysregulated, and time allocated for learning and creative activities is substantially encroached upon. Consequently, students may find it difficult to experience a sense of control and accomplishment in their academic pursuits and research, thereby suppressing their needs for autonomy and competence (Zhao and Kou, 2024). Furthermore, the social interactions cultivated by short video platforms are often superficial and symbolic, making it challenging to satisfy individuals’ need for genuine relatedness. When these three basic psychological needs are persistently unmet, an individual’s intrinsic motivation is likely to decline significantly. Intrinsic motivation serves as a critical driving force for stimulating and sustaining creativity. Creativity is not a passive occurrence but rather a process requiring active engagement, sustained exploration, and continuous trial and error. This process is highly dependent on the individual’s inherent interest and passion for the activity itself (Yuan et al., 2017). When college students lack intrinsic motivation for learning and creative activities, they are more inclined to choose passive entertainment over active creation, thereby missing opportunities to enhance their creativity (Goulet-Pelletier et al., 2023). It is noteworthy that the design of short video platforms themselves may also contribute to the erosion of users’ intrinsic motivation. Through algorithmic recommendations, immediate feedback loops, and intermittent reward mechanisms, these platforms orient user behavior toward the pursuit of external rewards, gradually diminishing users’ perception of the intrinsic value of the activities themselves (Zhang et al., 2023). This mechanism, whereby external rewards undermine intrinsic motivation, has been well-documented in psychological research. The present study extends this understanding to the context of short video addiction and creativity, further elucidating the profound impact of digital media on the psychological development of college students.

### 4.3 The mediating role of innovative self-efficacy

The mediating role of innovative self-efficacy in the relationship between short video addiction and creativity provides another important explanatory pathway in this study. The results indicated that short video addiction significantly and negatively predicted innovative self-efficacy, while innovative self-efficacy significantly and positively predicted creativity. This suggests that short video addiction can indirectly inhibit creative performance by diminishing individuals’ confidence in their own innovative capabilities. According to Social Cognitive Theory, self-efficacy is influenced by four primary sources of information: enactive mastery experiences, vicarious experiences, verbal persuasion, and physiological and affective states. Short video addiction may undermine these sources in several ways. First, at the level of enactive mastery experiences, excessive engagement with short videos leads to insufficient investment in academic and creative tasks, making it difficult for college students to accumulate successful innovation experiences. Instead, they are more prone to experiencing failure, which consequently lowers their self-evaluation of their own abilities (Mao and Liao, 2025). Second, concerning vicarious experiences, the content of short videos is predominantly entertainment-oriented and fragmented, lacking inspiring innovative role models and demonstrations of deep thought. Individuals thus find it difficult to obtain positive vicarious experiences from such content (Gao et al., 2023). Third, regarding verbal persuasion, short video use often encroaches upon time for real-life social interaction. As a result, individuals experience fewer in-depth conversations with peers and teachers, and opportunities to receive encouragement and constructive feedback are diminished. Finally, at the level of affective states, short video addiction is frequently accompanied by negative emotions such as anxiety, guilt, and self-reproach, which can further undermine an individual’s confidence in their own abilities. When an individual’s innovative self-efficacy remains persistently low, they are more likely to exhibit fear of difficulty, avoidance behaviors, and a tendency toward low effort when confronted with creative tasks, ultimately leading to poor creative performance (Li et al., 2020). It is important to note that innovative self-efficacy not only influences an individual’s willingness to engage in creative activities but also affects their persistence when encountering difficulties during such activities. Individuals with low self-efficacy are more prone to giving up when faced with setbacks, and this act of giving up can further reinforce negative evaluations of their own capabilities, thereby creating a vicious cycle (Oh and Pyo, 2023). Therefore, the negative impact of short video addiction on creativity, mediated by the weakening of innovative self-efficacy, may be characterized by its long-term and cumulative nature.

### 4.4 The chain mediating role of intrinsic motivation and innovative self-efficacy

The most significant finding of this study is the confirmation of the chain mediating role of intrinsic motivation and innovative self-efficacy in the relationship between short video addiction and creativity. This pathway elucidates how short video addiction indirectly affects creativity through a “motivation → belief” cognitive chain. Specifically, short video addiction first diminishes college students’ intrinsic interest in and willingness to engage in learning and creative activities, thereby depriving them of the motivational drive to participate in innovation. This decline in intrinsic motivation subsequently weakens their confidence in their own innovative capabilities, leading them to doubt their ability to successfully complete creative tasks at the belief level (Liu et al., 2025). Ultimately, this dual inhibition results in an overall decrease in creativity. This chain mechanism is fundamentally different from alternative explanations such as attention fragmentation. Attention fragmentation theory emphasizes that short videos directly impair sustained attention and deep processing at a cognitive level. In contrast, the “motivation → belief” pathway represents an indirect inhibitory process at the psycho-motivational level: short video addiction does not directly “deplete” cognitive resources; rather, it first erodes intrinsic interest (the motivational source) and then undermines confidence in one’s own innovative abilities (the belief support). Compared to the immediate effects of attention fragmentation, this chain pathway exhibits cumulative, lagged, and subtle characteristics. Thus, this mechanism complements, rather than replaces, attention fragmentation theory, collectively constituting a “dual pathway” – direct cognitive and indirect psycho-motivational – through which short video addiction affects creativity.

This chain mechanism suggests that motivation and belief play synergistic roles in individuals’ creative behavior: motivation provides direction and energy for action, while belief provides sustainability and resilience. When short video addiction erodes both of these psychological resources simultaneously, individuals’ creative behavior loses its essential support. Intrinsic motivation is a direct product of the satisfaction of basic psychological needs, whereas innovative self-efficacy is a cognitive representation of the successful experiences accumulated by individuals during the process of need satisfaction (Nong et al., 2023). When short video addiction hinders the fulfillment of basic psychological needs, the decline in intrinsic motivation represents an immediate response, while the decline in innovative self-efficacy is a longer-term cumulative outcome. Therefore, within the chain model, intrinsic motivation and innovative self-efficacy are not in a parallel relationship but exhibit a temporal sequence and logical progression. This finding also addresses the uncertainty in existing research regarding the relationship between these two constructs. Some studies suggest that intrinsic motivation is an antecedent of innovative self-efficacy, positing that only when individuals develop an interest in the activity itself are they willing to invest effort and attempt it, thereby accumulating experience and building confidence through these attempts (Gu et al., 2015). Other studies, conversely, argue that innovative self-efficacy reciprocally influences intrinsic motivation, suggesting that individuals only develop interest in an activity when they believe they can succeed in it (Xiang et al., 2023). The chain mediation model in the present study supports the former perspective, indicating that, within the context of the negative influence of short video addiction, the decline in intrinsic motivation is a more proximal psychological process, with the decline in innovative self-efficacy occurring as a subsequent consequence.

### 4.5 Theoretical contributions and practical implications

From a theoretical perspective, this study contributes to the existing literature in several ways. First, although previous research has examined the impact of digital media use on individual cognition, few studies have systematically investigated the relationship between the specific behavior of short video addiction and higher-order cognitive abilities such as creativity. Through empirical data, this study confirms that short video addiction exerts a significant negative predictive effect on creativity, thereby filling a gap in this research area and enriching the understanding of college students’ mental health and cognitive development in the digital age. Second, by incorporating both intrinsic motivation and innovative self-efficacy into the analytical framework, this study reveals their chain mediating role in the relationship between short video addiction and creativity. Compared with a single mediation model, the chain mediation model more comprehensively elucidates the dual “motivation → belief” pathway through which short video addiction inhibits creativity by eroding individuals’ intrinsic interest and self-belief, thereby deepening the theoretical explanation of this relationship. Furthermore, this study confirms the antecedent role of intrinsic motivation within the chain model, indicating that, under the negative influence of short video addiction, individuals first lose interest in creative activities and subsequently doubt their own abilities. This finding provides empirical evidence for clarifying the hierarchical relationship between motivation and self-efficacy in creative behavior, representing a theoretical contribution.

From a practical standpoint, this study offers actionable directions for intervention. First, regarding higher education institutions: Universities should strengthen the monitoring and guidance of students’ short video usage by implementing digital literacy education programs and establishing phone-free classrooms or screen-free periods to help students develop healthy digital media habits. Given the central role of intrinsic motivation, educators should adopt autonomy-supportive teaching styles and incorporate project-based or inquiry-based learning to enhance students’ autonomy and sense of competence. Furthermore, to boost innovative self-efficacy, universities should provide innovation competitions and research training camps that offer enactive mastery experiences, along with encouragement and recognition from teachers and peers. Second, regarding short video platforms: Platforms should reduce addictive design features such as infinite scrolling and auto-play, and incorporate health intervention tools like usage reminders and mandatory rest prompts. They may also explore innovation incentive models by promoting constructive content such as skill tutorials and creative challenges to redirect users from passive entertainment toward creative inspiration. Third, regarding families: Parents should model healthy screen use, create a low-distraction home environment, and establish a family screen time contract with daily viewing limits. Encouraging alternative activities like family discussions, shared reading, and hands-on creation, as well as providing affirmative communication, can help protect children’s self-worth. In summary, addressing short video addiction and its impact on creativity requires a multi-stakeholder effort: universities build supportive environments for deep thinking and innovation, platforms redesign their algorithms for social good, and families cultivate healthy digital habits at home. Collectively, these efforts can foster a digital ecosystem conducive to the creative development of college students.

### 4.6 Limitations and future directions

Although this study has made theoretical and empirical progress, several limitations should be acknowledged and addressed in future research. First, regarding research design, cross-sectional data were employed in this study, which precludes causal inferences among the variables. Although the chain mediation model was grounded in solid theoretical foundations, cross-sectional data cannot entirely rule out the possibility of reverse causality or the influence of third variables. Future studies could adopt longitudinal designs, tracking college students from their first to fourth years to examine the dynamic changes in short video usage behavior, intrinsic motivation, innovative self-efficacy, and creativity, thereby revealing the causal relationships and developmental trajectories over time. Additionally, experimental intervention studies could be introduced, designing interventions aimed at reducing short video use to observe their effects on intrinsic motivation, innovative self-efficacy, and creativity, thus providing more rigorous testing of causal pathways. Second, concerning sample selection, the sample of this study was primarily drawn from universities in Jiangsu Province, and there was a certain imbalance in the gender ratio, which may limit the generalizability of the findings. Future research should expand the sample coverage to include students from different regions, various types of institutions, and diverse academic backgrounds, thereby enhancing sample diversity and representativeness. Furthermore, attention should be paid to the moderating roles of demographic variables such as gender, grade level, and academic major in the model, to further explore group differences in the impact of short video addiction on creativity. Third, regarding measurement tools, all variables in this study were collected through self-report questionnaires, which may be subject to social desirability bias and common method bias. Although Harman’s single-factor test did not indicate severe common method bias, future research could incorporate multi-source data (e.g., teacher evaluations, peer nominations, objective behavioral records) or employ experimental tasks (e.g., divergent thinking tests) to measure creativity, thereby enhancing the objectivity and reliability of the data. Additionally, short video addiction could be validated using objective usage data (e.g., screen time, app usage frequency) to supplement subjective reports. Fourth, concerning the theoretical model, this study only examined the chain mediating roles of intrinsic motivation and innovative self-efficacy, without including other potential psychological mechanisms or moderating variables. Future research could further expand the theoretical framework by exploring the roles of factors such as emotional states, time management tendencies, social anxiety, and sleep quality in the relationship between short video addiction and creativity. Furthermore, personality traits (e.g., openness, conscientiousness), family environment (e.g., parenting styles), and school climate could be introduced as moderating variables to investigate the boundary conditions of the effect of short video addiction on creativity under different contexts. Fifth, regarding cultural context, this study was conducted within the Chinese cultural context, and the conclusions may not be directly generalizable to other cultural backgrounds. Differences may exist across countries in terms of short video usage habits, definitions of creativity, and evaluation criteria. Future research could conduct cross-cultural comparative studies to examine the similarities and differences in the relationship between short video addiction and creativity across different cultural contexts, as well as the cross-cultural consistency of the mediating mechanisms involving intrinsic motivation and innovative self-efficacy. Such efforts would provide more universally applicable theoretical guidance for digital media use and adolescent development in a globalized context.

## 5 Conclusion

Grounded in Self-Determination Theory and Social Cognitive Theory, a chain mediation model of the effect of short video addiction on college students’ creativity was constructed and validated in this study. The results indicated that short video addiction not only directly and negatively predicted college students’ creativity but also exerted inhibitory effects through three indirect pathways: the independent mediating role of intrinsic motivation, the independent mediating role of innovative self-efficacy, and the chain mediating role of “intrinsic motivation → innovative self-efficacy.” This finding reveals the dual “motivation → belief” pathway through which short video addiction inhibits creativity by eroding individuals’ intrinsic interest and innovative beliefs, thereby providing new empirical evidence for understanding the relationship between digital media use and higher-order cognitive abilities. The study confirms that short video addiction is a significant risk factor for college students’ creativity. Its negative impact is manifested not only through the direct consumption of cognitive resources but also indirectly through the weakening of individuals’ psychological motivational system. Intrinsic motivation and innovative self-efficacy exhibit a progressive relationship in this process: short video addiction first diminishes students’ intrinsic interest in creative activities, subsequently undermines their confidence in their own innovative capabilities, and ultimately leads to a decline in creativity. This finding contributes to clarifying the hierarchical relationship between motivation and self-efficacy in creative behavior. In summary, the negative impact of short video addiction on college students’ creativity is characterized by its multi-level and multi-path nature. Guiding college students to cultivate healthy digital media usage habits, while protecting and stimulating their intrinsic motivation and innovative self-efficacy, represents an important approach to promoting the healthy development of creativity.

## Data Availability

The original contributions presented in the study are included in the article/supplementary material, further inquiries can be directed to the corresponding author.
